# Clinicopathological Significance and Prognostic Value of Lactate Dehydrogenase A Expression in Gastric Cancer Patients

**DOI:** 10.1371/journal.pone.0091068

**Published:** 2014-03-07

**Authors:** Xuren Sun, Zhe Sun, Zhi Zhu, Haixia Guan, Junyan Zhang, Yining Zhang, Huimian Xu, Mingjun Sun

**Affiliations:** 1 Department of Gastroenterology, First Affiliated Hospital of China Medical University, Shenyang, Liaoning Province, China; 2 Department of Surgical Oncology, First Affiliated Hospital of China Medical University, Shenyang, Liaoning Province, China; 3 Department of Endocrinology and Metabolism, First Affiliated Hospital of China Medical University, Shenyang, Liaoning Province, China; 4 Department of Gastrointestinal Endoscopy, First Affiliated Hospital of China Medical University, Shenyang, Liaoning Province, China; The Chinese University of Hong Kong, Hong Kong

## Abstract

**Introduction:**

LDH-A, the enzyme responsible for transforming pyruvate into lactate, has been demonstrated to be up-regulated in many types of cancer and to give rise to more aggressive behavior by regulating proliferation and anti-apoptosis. However, its expression in gastric cancer (GC) has not been characterized thoroughly. The purpose of this study was to clarify the expression and potential impact of LDH-A in GC.

**Methods:**

We examined LDH-A expression by immunohistochemistry on GC tissue microarray (TMA) and using Western blot on fresh GC tissues and cell lines. Prognostic value and correlation with other clinicopathologic factors were evaluated. We transfected siRNA into GC cells against LDH-A. LDH-A was analyzed by Western blotting and real-time RT-PCR. Cell growth was evaluated in vitro and in vivo. Lactate and ATP production by cells were determined.

**Results:**

There was significantly higher LDH-A expression in carcinoma than in non-neoplastic mucosa (NNM). There was a positive correlation of LDH-A expression with age, histological type and Lymph node metastases. Survival analysis demonstrated that high expression of LDH-A in GC was associated with lower overall survival (OS). When stratified by Lauren grade and histological classification, significance appeared in diffuse type and undifferentiated type GC. In multivariate analysis, the LDH-A expression in GC was an independent prognostic risk factor for OS (hazard ratio = 1.829, 95%CI 1.375–2.433,P<0.0001). Specific siRNA against LDH-A in GC cell line retarded cell growth both in vitro and in mouse models. LDH-A knockdown also reduced lactate and ATP production in GC cells.

**Conclusions:**

Our study indicated the oncogenic role of LDH-A in GC. LDH-A expression is an independent prognostic risk factor in GC patients and up-regulated expression of LDH-A could be predictive of poor outcomes in diffuse type and undifferentiated type GC. Our results suggested that LDH-A might be a potential therapeutic target in gastric cancer.

## Introduction

The metabolic properties of cancer cells diverge significantly from those of normal cells, as cancer cells preferentially utilize glycolysis instead of mitochondrial oxidative phosphorylation even in the presence of oxygen; this phenomenon is known as the Warburg effect [1] [2], [3]. The surprisingly high rate of taking up glucose and lactate production in tumors in the presence of oxygen led Warburg to speculate that aberrant metabolism could be the cause of many cancers [2]. However, the advantage of the metabolic transformation confers to cancer cells remains unclear [4], [5]. Recently, the discovery of the connection between oncogenes and metabolic processes has led to a resurgence of interest in Warburg’s work. There is increasing evidence indicating that the adaption of aerobic glycolysis by cancer cells might facilitate cancer proliferation [5]. Cancer metabolism has been under extensive exploration in the hope of discovering new effective therapies for cancer.

LDH-A, is a crucial enzyme that plays an important role in the final step of the Warburg effect by converting pyruvate to lactate. In various types of human cancers, the expression of LDH-A was up-regulated [6], [7]. In esophageal squamous cell carcinoma, LDH-A was up-regulated in cancer tissues and promoted the survival of tumor cells [8]. Additionally, LDH-A was reported to enhance the growth and migration of gastric cancer cells [9]. However, the expression status and the function of LDH-A in gastric cancer(GC) still remain unknown.

In this study, we examined the expression of LDH-A in a large cohort of GC specimens and correlated its expression with clinical pathological parameters and overall survival (OS). Our results demonstrate that the expression of LDH-A was up-regulated in the clinical gastric cancer samples, and protein expression was correlated to age and histological classification. In addition, we found that high LDH-A expression was associated with worse prognosis in GC patients. Taken together, our study revealed the oncogenic function of LDH-A in gastric cancer and suggested LDH-A as a new potential prognostic factor and a potential therapeutic target.

## Materials and Methods

### Patients

The present study included 264 patients with GC who followed curative surgery from January 2006 to May 2007 at the First affiliated hospital of China Medical University. The group was composed of 194 males and 70 females with a mean age of 59 (range, 29–81) years. None of the patients underwent chemotherapy or radiotherapy before surgery. Follow-up information had been collected from all patients.

### Ethics Statement

Ethical approval for this research was obtained from the Research Ethics Committee of China Medical University, China. All patients providing tumor tissue as well as normal gastric tissue samples signed a consent form prior to surgical removal of the gastric carcinoma to allow for this research to be undertaken. All of the animal study procedures were in accordance with the National Institutes of Health Guide for the Care and Use of Laboratory Animals and were performed according to the institutional ethical guidelines. These laboratory animals were purchased from Shanghai Branch Center, Institute of Laboratory Animal Science, Chinese Academy of Medical Science (also known as Shanghai CAS, certificate number: SCXK [Hu] 2003-0003). All experimental procedures were conducted in conformity with institutional guidelines for the care and use of laboratory animals, and protocols were approved by the Institutional Animal Care and Use Committee in China Medical University, Shenyang, China. All efforts were made to minimize suffering.

### Tissue Samples and Pathology

All patient-derived paraffin embedded Gastric cancer specimens (n = 264) and their matched NNM specimens(2 cm away from the carcinoma n = 209) were collected from surgical resection and archived under protocols approved by the Institutional Review Boards of the First affiliated Hospital China Medical University. Fresh gastric carcinoma and adjacent NNM were collected and frozen in −80°C until protein extraction by homogenization in RIPA lysis buffer. The histologic diagnosis and other microscopic characteristics were confirmed by pathologists and the TNM staging for each gastric carcinoma was evaluated according to the Union International Contre le Cancer (UICC) system for the extent of tumor spread. Histologic architecture of gastric carcinoma was expressed in Lauren’s classification and the World Health Organization (WHO) classification. Furthermore, tumor size, depth of invasion, and lymphatic were determined.

### Cell Lines and Culture

The human gastric cancer cell lines MKN28 (well differentiated), AGS, SGC-7901 (moderately differentiated), MGC803 (poorly differentiated) and the human normal gastric cell line GES-1 purchased from the cell bank of Chinese Academy of Sciences. Five cell lines were maintained in RPMI 1640 (Hyclone, Logan city, USA) supplemented with 10% fetal bovine serum (FBS). All the cell lines were in a 5% CO_2_ humidified atmosphere at 37°C.

### Tissue Microarray (TMA) and Immunohistochemistry

Representative areas of solid tumors and adjacent NNM were identified in HE-stained sections of the selected cases and a 1.5-mm-diameter tissue core per donor block was punched out and transferred to a recipient block with a maximum of 200 (10×20) cores using a 1.5-mm diameter punch instrument. After re-melted, sections (4 µm-thick) were consecutively cut from each tissue microarray block, HE staining was performed on TMA for confirmation of tumor and mucosa tissue. Immunohistochemical analysis was performed on TMA sections, Pressure cooker mediated antigen retrieval was performed in citrate buffer (pH 6.0) for 10 min. Sections were incubated with 1∶300 dilution of LDH-A (muscle subunit) Rabbit Monoclonal Antibody (epitomics) overnight at 4°C, and then incubated with goat anti-mouse or anti-rabbit Envision System Plus-HRP (Dako Cytomation) for 30 min at room temperature. After rinsing three times in PBS for 10 min each, the sections were incubated with DAB for 1 min, counterstained with mayer hematoxylin, dehydrated, cleared and mounted. Omission of the primary antibody was used as a negative control.

### Western Blot Analysis

Proteins concentrations of samples extracted from tissue specimens and cell lines using radioimmune precipitation assay lysis buffer were determined using Bradford reagent (Sigma) according to the manufacturer’s instructions. Denatured protein was separated on an sodium dodecyl sulfate (SDS)-polyacrylamide gel (10% acrylamide) and transferred to polyvinylidene fluoride membranes (Millipore, Bedford, MA), which was then blocked in 5% fat-free milk in Tris-buffered saline (pH 7.5). For immunoblotting, the membrane was incubated overnight with LDH-A (muscle subunit) Rabbit Monoclonal Antibody (1∶1500)(epitomics) at 4°C. Then, it was rinsed by TBST and incubated with secondary antibodies for 15 minutes. Signals were detected by enhanced chemiluminescence (Pierce, Rockford, IL).

### Follow-up after Surgery

The 264 patients who underwent gastroctomy were subjected to close clinical observation, including chest/abdominal/pelvic computed tomographic (CT) imaging, CEA level, and blood testing at 2 to 3 month intervals and a yearly gastroscopy. Follow-up was in accord with National Comprehensive Cancer Network (NCCN) Practice Guidelines in gastric cancer. Overall survival (OS) rate was defined as the interval from the initial surgery to death. The end date of the follow-up study for conducting the analysis was June 29, 2012.

### Evaluation of Immunohistochemical Staining

Immunoreactivity was evaluated independently by two researchers who were blinded to patient outcome. The evaluation was based on the staining intensity. Staining intensity for LDH-A was scored as 0 (negative); 1 (weak); 2 (moderate); and 3 (strong). The specimens were divided into two groups according to their scores: 0 and 1 are low expression group; 2 and 3 are high expression group. In the event of a discrepancy in scoring, the slides were re-examined by both pathologists under a microscope.

### Generation of Small Interfering (si) RNA Knockdown Stable Cell Lines

Based on the LDH-A sequence, shRNA was designed using siRNA Target Finder (Ambion): The nucleotide sequence of the inserted ShLDH-A was 5′-ATCCAGTGGATATCTTGACCTACG TGGCT-3′. The cell line generated by infecting cells with scrambled plasmid was used as a control. 10 µg shRNA and 25 µl Lipofectamine 2000 (Invitrogen Life Technologies, Carlsbad, CA, USA) were mixed together with 1350 µl RPMI-1640 medium (without FBS), and then the mixture was transfected into MGC-803 gastric cancer cells. The mixture was added into a 25-cm^2^ culture flask that was previously plated with 1×10^6^ MGC-803 gastric cancer cells. The culture medium was replaced with complete RPMI-1640 medium 6 h following inoculation and at 28 h after transfection, stable cell clones were selected for 2 weeks with neomycin and analyzed by immunoblotting.

### RNA Extraction and Real-time Quantitative RT-PCR Analysis

Total RNA was isolated from cells using TRIzol reagent according to the instructions of the manufacturer (Invitrogen), and 1 µg of RNA was processed directly to cDNA using a reverse transcription kit (Promega, Madison, Wisconsin), according to the manufacturer’s instructions. Amplification reactions were performed in a 20-µl volume with 0.2 µl of SYBR Green (Bio-Rad, Hercules, California). These reactions were performed in triplicate using a BioRad iCycler (Bio-Rad). β-Actin was used as an internal control. The primers used were as follows:LDH-A, 5′-CTCCTGTGCAAAATGGCAAC-3′(forward) and 5′-CCTAGAGCTCACTAGTCACAG-5′(reverse); andβ-Actin, 5′-GATCATTGCTCCTCCTGAGC-3′ (forward) and 5′-ACTCCTGCTTGCTGATCCAC-3′ (reverse). The specificity of real-time quantitative PCR was verified by melting curve analysis and agarose gel electrophoresis.

### MTT and Colony Formation Assay

For cell growth analysis, an equal number of cells were seeded in 48-well plates. The total number of cells was measured every day by the MTT assay according to the manufacturer’s protocol(Roche Applied Science). For colony formation analysis, equal number of control cells and cells silencing the expression of LDH-A were seeded into 60 mm culture dish in triplicate. Cells were cultured for 7 days, Cell growth was stopped after 7 days in culture by removing the medium and adding 0.5% crystal violet solution in 20% methanol. After staining for 5 min, the fixed cells were washed with phosphate-buffered saline (PBS), photographed, and dissolved with 1% SDS. The number of colonies in each group was counted.

### Tumorigenicity Assay

The MGC-803 GC cells xenografts were produced on 6-week-old female athymic nude mice (Vital River Laboratories, Beijing, China) by injection of 5×10^6^ cells in 0.2 mL of PBS into the flank of each mouse (6 mice/group). Tumor formation was assessed every week. Tumor volume was calculated according to the formula: V (mm^3^) = a×b^2^/2 (where a is the largest superficial diameter and b is the smallest superficial diameter).

### Measurement of Lactate Concentration and ATP Production

After 48 hours of incubation, lactate, and ATP levels in the culture media were measured using commercially available assay kits (purchased from BioVision [Mountain View, California], and Roche Applied Science, respectively). Results were corrected for the final cell count number.

### Statistical Analysis

All statistical analyses were performed by using the SPSS 17.0 (SPSS Inc, Chicago, IL). Overall survival rates were determined using the Kaplan–Meier estimator, an event being defined as death for cancer-related cause. The log-rank test was used to identify differences between survival curves. In univariate analysis, two-tailed χ^2^ tests or two-tailed t test were used for statistical comparisons. And Cox’s proportional-hazard model was used in univariate analysis and multivariate analysis, to identify significant factors correlated with prognosis. For all analyses, only P values<0.05 were considered significant.

## Results

### LDH-A Expression in Human Gastric Cancer Tissue

**Figure 1:**
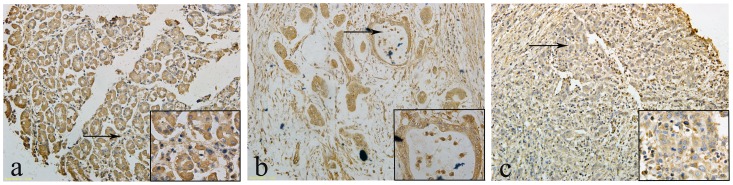
Immunohistochemical staining of LDH-A in gastric samples. LDH-A was expressed in the cytoplasm of NNM and carcinomas: **a** LDH-A positivity was observed in the cytoplasm of NNM, **b** LDH-A was detected in the cytoplasm of differentiated gastric carcinoma, **c** and in the poorly-differentiated gastric carcinoma.

**Figure 2:**
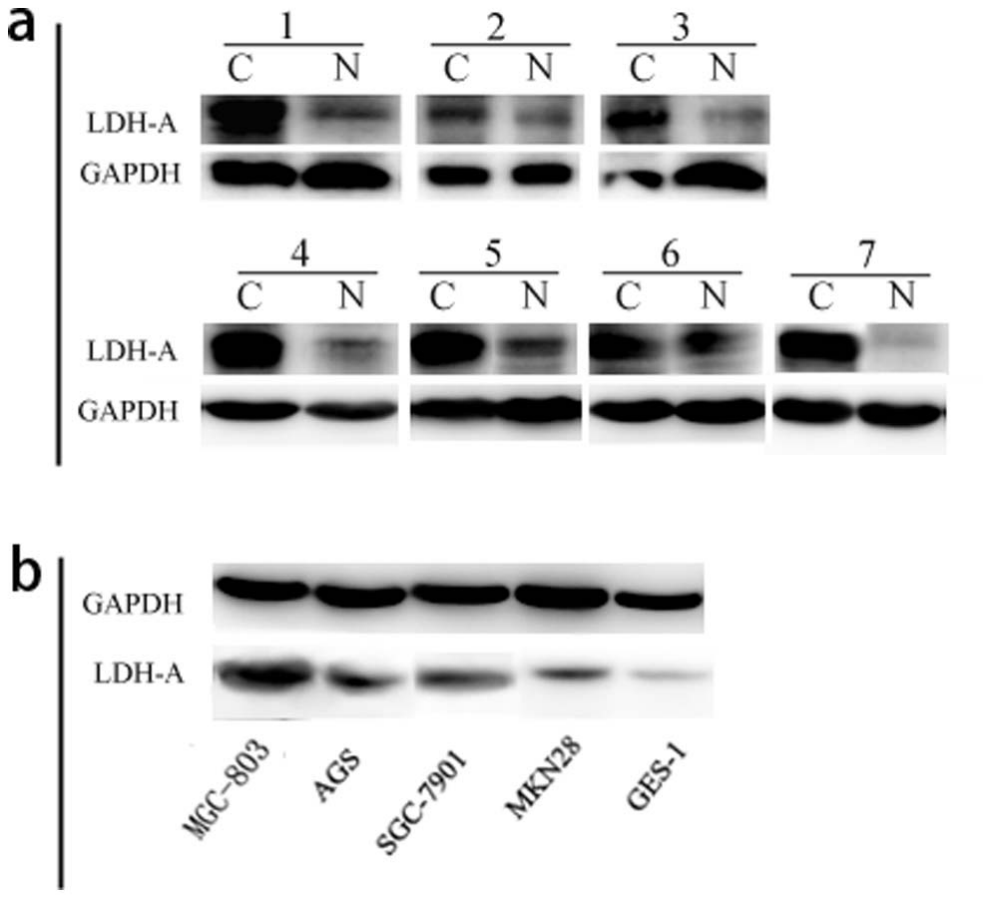
Up-regulation of LDH-A in GC tissues and cell lines was confirmed by western blot. **a** The protein level of LDH-A in gastric cancer samples and paired normal tissues was analyzed. **b** Expression of LDH-A in 4 gastric cancer cell lines and GES-1 was determined. The LDH-A expression in the GC cell lines MGC803 and AGS was up-regulated compared with GES-1. GAPDH was used as an internal control.

We graded stained sections of TMAs of GC and NNM tissue cores for their cytoplasmic immunohistochemical staining intensity against LDH-A protein. The readable samples included 264 gastric carcinoma and 209 matched NNM. The typical diffuse cytoplasmic staining of the protein can be found in many gastric carcinoma and normal gastric tissues, as shown in Figure 1, cancer tissue showed positive cytoplasmic staining for 201 cases (76.14%), while among 209 cases of NNM, 131 cases (62.68%) had LDH-A positive expression (Table 1). To investigate the expression quantity of LDH-A in GC samples, the expression of LDH-A was examined by western blot analysis in seven randomly selected tumors and the paired normal tissues. Up-regulation of LDH-A was observed in GC samples compared with the paired normal tissues (Fig. 2a). These studies indicated that LDH-A was significantly upregulated in cancerous specimens compared to adjacent non-cancerous tissues (Table 1. p = 0.002).

**Table 1:** Expression of LDH-A in NNM and cancerous tissues.

Tissue Sample	LDH-A expression				P
	n	Low	High	PR(%)	
Adjacent Normal Mucosa	209	78	131	62.68	0.002
Primary Cancer Tissue	264	63	201	76.14	

### Expression Analysis of LDH-A in Gastric Cell Lines

Western blotting analysis was performed in gastric cell lines. The western blot showed that expression of the LDH-A was significantly strong in poorly differentiated MGC803 and moderately differentiated AGS; weak in moderately differentiated SGC-7901 and well differentiated MKN28; and absent in human normal gastric cell line GES-1 (Fig. 2 b).

### Clinicopathological Variables in 264 Cases of Gastric Carcinoma

As summarized in Table 2, We found that increased expression of LDH-A was significantly associated with age of patients (P = 0.004) and histological type (P = 0.02), lymph node metastasis (P = 0.043), but not with gender (P = 0.072), tumor size (P = 0.086), depth of invasion (P = 0.328), lymphatic invasion(P = 0.738), lauren grade (P = 0.152) or TNM stage (P = 0.417).

**Table 2:** Relationship between LDH-A expression and clinico-pathologic features of GC.

Clinicopathologic	Cases	LDH-A expression			
Features variables		−	+	PR (%)	P value
	264	63	201	76.14	
Gender					0.072
Female	70	11	59	84.29	
Male	194	52	142	73.2	
Age(years)					0.004
<60	133	42	91	68.42	
≥60	131	21	110	83.97	
Tumor size (cm)					0.086
<4	81	25	56	69.14	
≥4	183	38	145	79.23	
Depth of invasion					0.328
T_is-1_	25	8	17	68	
T_2–4_	239	55	184	76.99	
Histological type					0.020
Differentiated	119	20	99	83.19	
Undifferentiated	145	43	102	70.34	
Lauren grade					0.152
Intestinal type	130	26	104	80.00	
Diffuse type	134	37	97	72.39	
TNM Stage					0.417
0–I	38	11	27	71.05	
II–IV	226	52	174	76.99	
Lymph node metastases					0.043
Negative	80	26	54	67.50	
Positive	184	38	146	79.35	
Lymphatic invasion					0.738
Absent	199	49	150	75.38	
Present	65	14	51	78.46	

### Survival Analysis

**Figure 3:**
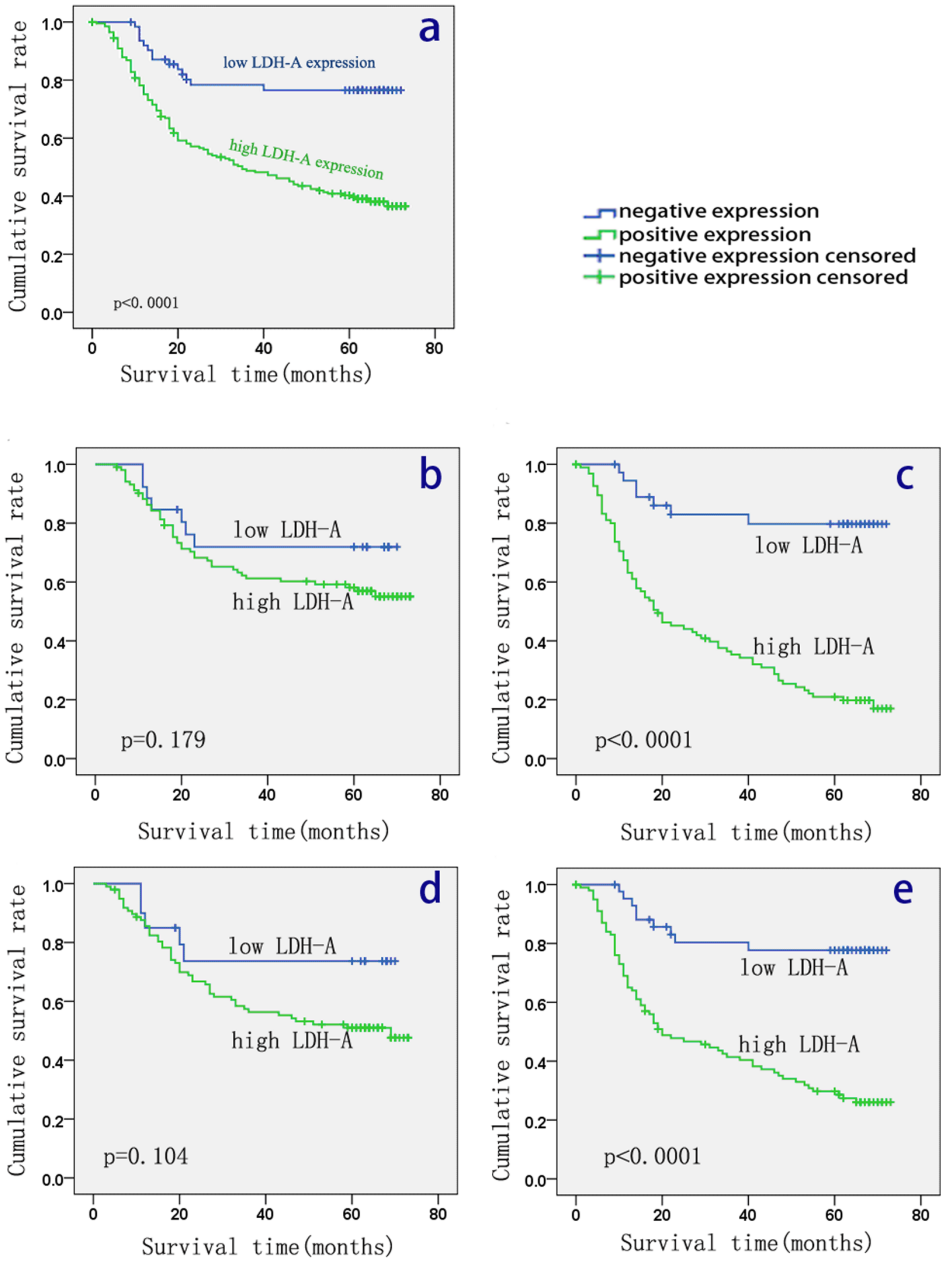
Correlation between LDH-A expression and prognosis of patients with GC. Kaplan-Meier curves for cumulative survival rate of patients with GC according to GC tissue LDH-A expression(a), Kaplan-Meier curves for cumulative survival rate of patients with intestinal-type GC(b) and diffuse-type GC(c) stratified by the Lauren grade, and Kaplan-Meier curves for differentiated type GC(d) and undifferentiated type GC(e) stratified according to Histological type.

The 5-year OS rate of the 264 patients with primary gastric cancer was 46% (122/264), with 142 deaths observed during the follow-up period. The median duration of follow-up was 50 months (range, 9–78 months). Survival analysis by Kaplan-Meier survival curve and log-rank test demonstrated that patients with high expression of LDH-A in tumor tissue had a significantly worse overall survival than patients with tumor low LDH-A expression (P<0.001; Fig. 3). With the purpose of exploring the potential relationship between the expression of LDH-A and the prognosis in different types of GC, we assessed the survival of patients in different subgroups: When statistical analysis was performed stratified by Lauren grade and histological classification, significance appeared in diffuse type GC and undifferentiated type GC (P<0.001, respectively), but not in intestinal (P = 0.179) or differentiated (P = 0.104) ones. Multivariate analysis was performed using the Cox proportional hazards model for all of the significant variables in the univariate analysis. Table 3 shows the significance: Adjusted for other covariates, the results from the multivariate analysis showed that LDH-A expression (P<0.001), lymphatic invasion (P = 0.046) and Lauren grade (P = 0.001), TNM stage (P = 0.006), but not age (P = 0.053), tumor size (P = 0.111), depth of invasion (P = 0.598), lymph node metastasis (P = 0.089), or histological type (P = 0.674), were independent prognostic risk factors for OS (Table 3).

**Table 3:** Univariate and multivariate analysis for overall survival after surgery using cox RR relative risk.

Variables	Univariate analysis		Multivariate analysis	
	HR(95%CI)	P value	HR(95%CI)	P value
LDH-A expression				
Positive/Negative	1.853 (1.404–2.444)	<0.001	1.829 (1.375–2.433)	<0.001
Age				
<60/≥60	1.446 (1.030–2.032)	0.033	1.431(0.995–2.058)	0.053
Tumor size				
<4/≥4 cm	2.297 (1.505–3.504)	<0.001	1.429 (0.921–2.217)	0.111
Depth of invasion				
T_is-1_/T_2–4_	5.114(1.889–13.843)	0.001	1.414(0.389–5.137)	0.598
Lauren grade				
Intestinal/Diffuse	1.978 (1.396–2.803)	<0.001	2.142 (1.350–3.399)	0.001
TNM Stage				
0–I/II–IV	8.235 (3.042–22.30)	<0.001	1.410(1.105–1.799)	0.006
Lymph node metastasis				
Positive/Negative	3.146 (1.989–4.977)	<0.001	1.571 (0.934–2.644)	0.089
Lymphatic invasion				
Positive/Negative	2.041 (1.424–2.924)	<0.001	1.467 (1.006–2.139)	0.046
Histological type				
Differentiated/Undifferentiated	1.460 (1.033–2.063)	0.032	0.902 (0.579–1.404)	0.674

### Knockdown of LDH-A by siRNA Reduces the Expression of LDH-A in MGC-803

**Figure 4:**
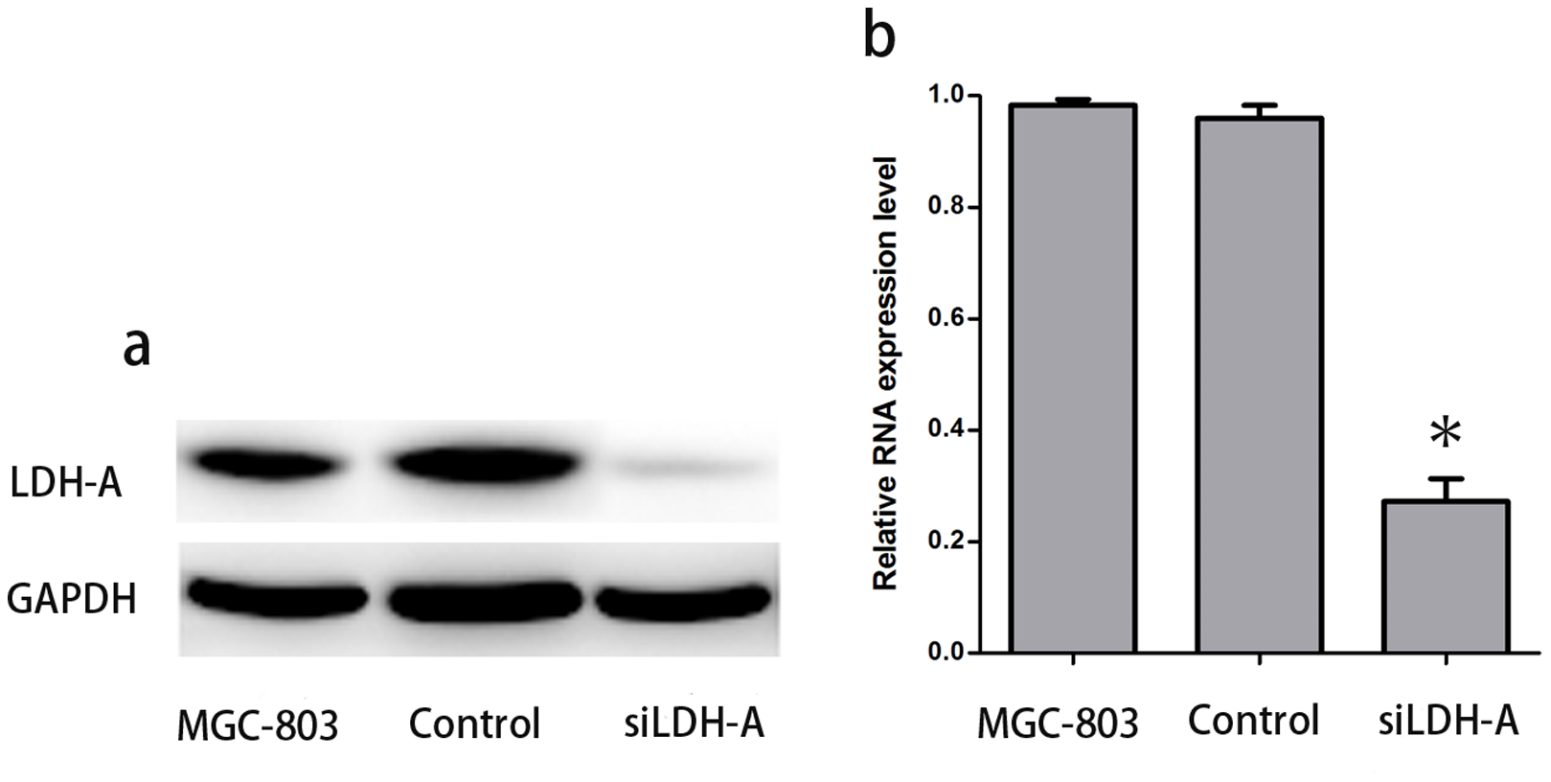
Effect of siLDH-A on the expression of LDH-A in GC cell line MGC-803. In the LDH-A-siRNA expressing cells, expression of LDH-A protein assayed by western blot was hardly detectable(a); Significant down-regulation of LDH-A at the mRNA level was detected after siLDH-A infection(b).

According to the western blot results and real-time RT-PCR, the expression of LDH-A decreased effectively in the LDH-A-siRNA expressing MGC-803 cells. As shown in Figure 4, expression of LDH-A protein assayed by western blot was hardly detectable, and the PKM2 mRNA level was reduced by 74% in the LDH-A-siRNA expressing MGC-803 cells. LDH-A expression remained unaltered in both GC cell lines and the cell lines expressing a control siRNA.

### Knockdown of LDH-A Inhibited the Growth of GC Cell Lines MGC-803

**Figure 5:**
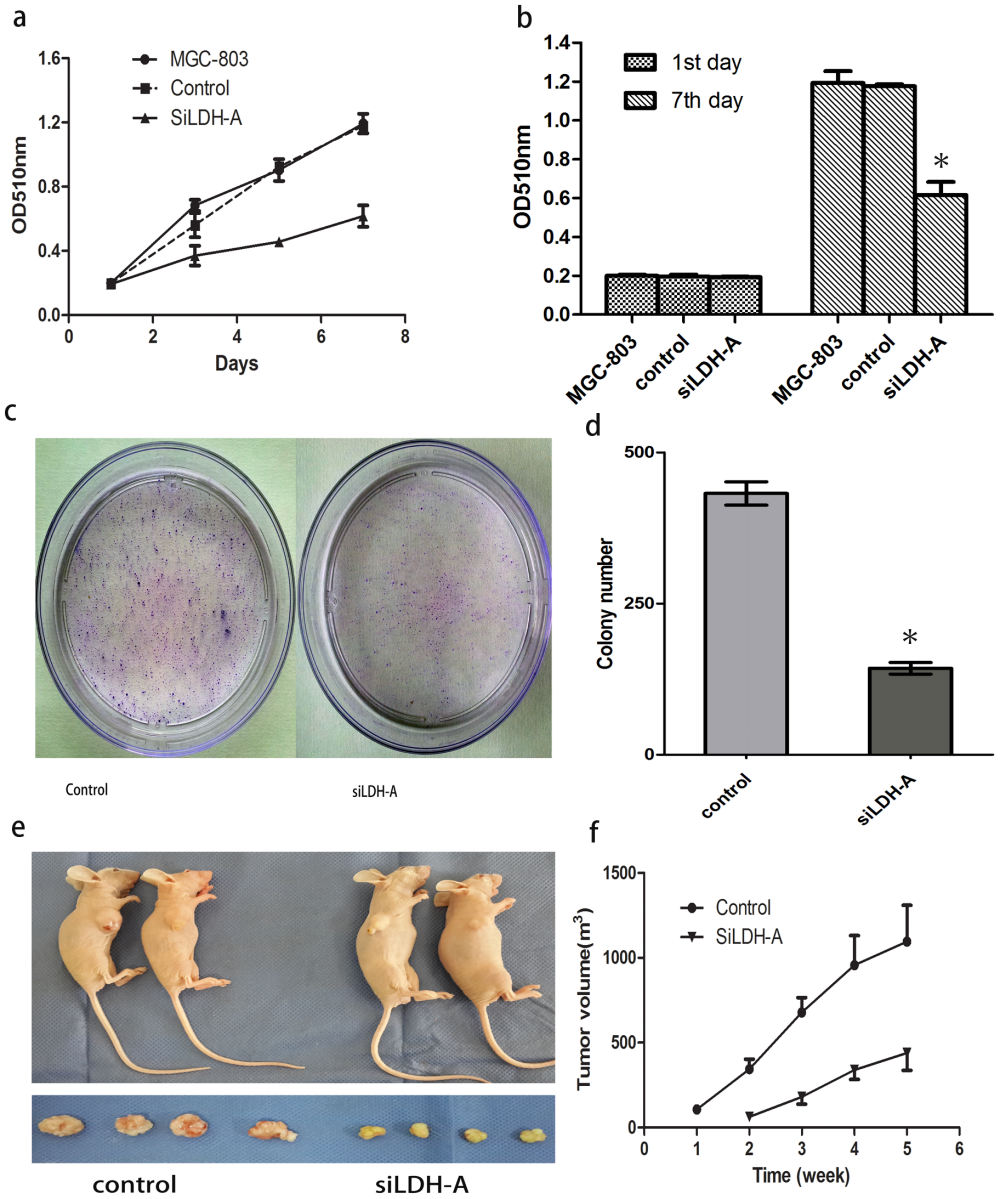
Inhibition of cell growth by siRNA knockdown of LDH-A in human GC cell line MGC-803. The growth of cells expressing siRNA were decreased by 64%, after 168 hours of incubation compared with that of the control cells in MTT assay(a and b, P<0.05). Silencing the expression of LDH-A attenuated the colony formation of MGC-803 cells (c and d). The in vivo tumorigenic capacity of MGC-803 cell line was reduced significantly by siRNA knockdown of LDH-A(e and f, p<0.05).

To assess tumor cell growth, we used MTT and colony formation assays. In the MTT assay, silencing the expression of LDH-A inhibited the proliferation of MGC-803 cells (Fig. 5a,b). The growth of MGC-803 cells expressing LDH-A siRNA decreased by 64%, after 168 hours of incubation compared with that of the control cells (P<0.05). Consistently, silencing the expression of LDH-A attenuated the anchorage-independent growth of MGC-803 cells (Fig. 5c, d). LDH-A knockdown in these cells prevented colony formation, indicating that anchorage-dependent growth was restored. These results further suggested the oncogenic role of LDH-A in GC cancer.

### Knockdown of LDHA Inhibited the Tumorigenicity in Xenograft Mouse Models

In this study, we examined the tumorigenic capacity of LDH-A in vivo. The tumorigenicity in vivo of MGC-803 siLDH-A cells and control cells was assessed by sc injection of cells in athymic nude mice. Consistent with the in vitro studies, silencing the expression of LDH-A dramatically attenuated the tumorigenicity of LDH-A cells in tumor size. For all matched xenografts, siLDH-A tumors were significantly smaller than those of the controls (Fig. 5e,f).

### Knockdown of LDH-A by siRNA Reduces Lactate and ATP Production in Human GC Cell Lines MGC-803

**Figure 6:**
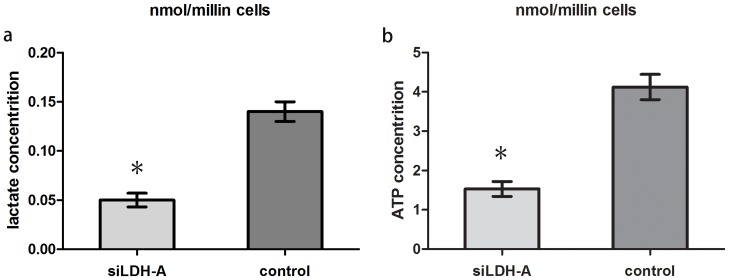
Knockdown of LDH-A in GC cell line MGC-803 reduced lactate and ATP production. After 48 hours of incubation, LDH-A knockdown led to significantly reduced lactate(a) and ATP production(b) in MGC-803 cells(p<0.05).

Because LDH-A is the key enzyme for transforming pyruvate into lactate, alteration of its expression in GC cells should affect lactate production and energy metabolism. To test this hypothesis, we compared lactate and ATP production between LDH-A siRNA knockdown cells and control cells after a 48-hour incubation. When LDH-A was knocked down by siRNA, the ability of the cell to produce lactate and ATP was dramatically decreased by 61.5% and 63.3% at 48 hours, respectively(Fig. 6a,b).

## Discussion

In the 1920s, Warburg first demonstrated that cancer cells take up higher rates glucose than normal cells and produce energy almost by aerobic glycolysis for their energy requirement to adapt environmental constraints such as intermittent hypoxia [10], [11]. However, this metabolic change is not simply an adaptation to a hypoxic environment but is an active cellular strategy that confers a significant advantage for proliferation and malignancy. Now Vegran *et al*. [12] have reported that lactate can directly modulate the phenotype of endothelial cells, potentially representing an important mechanism by which tumors control their own blood supply via vascular morphogenesis. Because reactive oxygen species (ROS) are natural by-products of mitochondrial respiration, it has been proposed that the conversion of glucose to lactate could protect cancer cells from oxidative stress [13]. Recently, findings suggest that reinstating normal oxidative phosphorylation in cancer cells may not only inhibit cell growth and proliferation but also impair the metastatic capacity of malignant cells [14]. Therefore, the importance of constitutively up-regulated aerobic glycolysis has recently been recognized in tumor progression, but the molecular mechanisms leading to this phenotype and their contributions to cancer development are not well understood.

LDH-A, the enzyme responsible for transforming pyruvate into lactate, plays an important role in the process of glycolysis. Several research have reported that the expression of LDH-A could be induced by a number of oncogenes [15]. These reports support the idea that LDH-A induction is critical for the oncogenic activity of these oncogenes in cancer cells.

The oncogenic activity of LDH-A has been reported in esophageal carcinoma, pancreatic cancer and gastric cancer cells [8], [9], [16]. Yongjie Zhang *et al*. reported that LDH-A reduction can suppress the tumorigenicity of intestinal-type gastric cancer (ITGC) cells by downregulating Oct4 [17]. And ZhiYu Wang *et al*. reported that LDH-A silencing suppresses breast cancer tumorigenicity through induction of oxidative stress mediated mitochondrial pathway apoptosis [18]. Here, we examined formalin-fixed, paraffin-embedded tumor tissue from 264 patients for the expression of LDH-A and reported the characterization of LDH-A expression in human GC, and present its correlation with clinicopathological parameters and patients’ prognosis. First, we found that the expression of LDH-A was elevated in GC samples compared with the matched normal tissues. This result may be consistent with previous study that in various types of human cancers, the expression of LDH-A was up-regulated [4], [5]. In tumor tissue, our study showed that LDH-A expression was strongly correlated with GC clinicopathologic characteristics: age, lymph node metastasis and histologic classification. So we propose that the up-regulated LDH-A expression may contribute to proliferation and the development of GCs. Second, we further analyzed the prognostic role of LDH-A on overall survival of patients with GC. Kaplan-Meier analysis showed a significant association between LDH-A expression and overall survival of patients, and patients with stronger LDH-A staining had a lower survival rate. When statistical analysis was performed stratified by Lauren grade and histological classification, significance appeared in diffuse type GC and undifferentiated type GC, but not in intestinal or differentiated ones. Specific siRNA against LDH-A was performed in MGC803 cells and retarded cell growth both in vitro and in mouse models. LDH-A knockdown also reduced lactate and ATP production in GC cells. Therefore, our results suggest that expression of LDH-A was an independently prognostic factor of OS, and indicated that LDH-A might have an oncogenic role in tumor development, especially in diffuse type GC or undifferentiated type GC patients. Consistent with our study, it has been reported that knocking down the expression of LDH-A in human hepatocellular carcinoma cells could induce apoptosis [8], and downregulation of LDH-A could increase the production of ROS and cytosolic Ca2+ signaling, which decreased the inner mitochondrial membrane potential and led to the release of cytochrome C in hepatocellular carcinoma cells.

Taken together, our study indicates that up-regulated expression of LDH-A provides gastric cancer cells with growth advantages. At the same time, our study suggests LDH-A as a potential therapeutic target.
