# Expression of Concern: Clinicopathological Significance and Prognostic Value of Lactate Dehydrogenase A Expression in Gastric Cancer Patients

**DOI:** 10.1371/journal.pone.0273730

**Published:** 2022-08-23

**Authors:** The PLOS ONE Editors

After this article [1] was published, a question was raised about how the normal tissue was obtained; subsequent editorial assessment identified text similarities and issues related to the western blot images in Figs 2 and 4. Specifically:

- There are regions of text similarity, particularly in the Discussion section, between this article [1] and the previously published article [2].
- In Fig 2A, there appear to be vertical discontinuities between the bands in the LDH-A blots in panels 5 and 7.
- In Fig 2B, there appear to be vertical discontinuities between lanes 2 and 3 and between lanes 3 and 4 in the LDH-A blot.
- In Fig 4A, there appears to be a vertical discontinuity between lanes 2 and 3 in the LDH-A blot.

In editorial follow up on these issues, the corresponding author stated that the molecular weights of LDH-A and GAPDH are very close, and the western blot bands would overlap or have unclear boundaries. They therefore adopted a method of separating the target protein (LDH-A) and internal reference protein (GAPDH) under the same conditions, which the corresponding author stated could cause the appearance of vertical discontinuities. An Academic Editor reviewed the figures and advised that running different gels under the same conditions for the target protein and the reference protein is not considered appropriate experimental design, and does not explain the vertical discontinuities within the LDH blots.

Additionally, in editorial follow up, the corresponding author also stated that their samples were from the surgically resected tissues of patients with gastric cancer and that the normal tissue samples were from greater than 2 cm from the cancerous tissue. The consulting Academic Editor advised that experimental approaches are preferable to demonstrate the normality of these cells.

During editorial follow up, it came to light that one of the cell lines used in this article [1], MKN28, described as a human gastric cell line in the article [1], was identified as a contaminated cell line prior to publication [3]-[4], and has been shown to be a MKN74 derivative, also a human gastric cell line [5]. It also came to light that two other cell lines used in this article [1], SGC-7901 and MGC803, also described as human gastric cell lines in the article [1], have subsequently been identified as contaminated cell lines, and have been shown to be a HeLa derivative (SGC-7901) [6–9] and a HeLa derivative with another cell of unknown origin respectively (MGC803) [10].

It appears that reference 8 in article [1] may have been cited in error in one instance, near the end of the Discussion section. Reference 8 in article [1] refers to esophageal squamous cell carcinoma, rather than hepatocellular carcinoma cells as mentioned in this section of the text in article [1].

The underlying study data and approval documentation have not been provided and the first author has indicated that the delay in accessing the requested material is due to COVID measures affecting the laboratory.

In light of the above issues with Figs 2 and 4A, which could not be resolved in the absence of the original underlying data, the *PLOS ONE* Editors issue this Expression of Concern.
